# Prediction of host-pathogen protein interactions by extended network model

**DOI:** 10.3906/biy-2009-4

**Published:** 2021-04-20

**Authors:** İrfan KÖSESOY, Murat GÖK, Tamer KAHVECİ

**Affiliations:** 1 Department of Computer Engineering, Faculty of Engineering, Yalova University, Yalova Turkey; 2 Department of Computer and Information Science and Engineering, University of Florida, Gainesville, FL USA

**Keywords:** Infectious diseases, host-pathogen interactions, protein-protein interactions, protein networks, machine learning, bioinformatics

## Abstract

Knowledge of the pathogen-host interactions between the species is essentialin order to develop a solution strategy against infectious diseases. In vitro methods take extended periods of time to detect interactions and provide very few of the possible interaction pairs. Hence, modelling interactions between proteins has necessitated the development of computational methods. The main scope of this paper is integrating the known protein interactions between thehost and pathogen organisms to improve the prediction success rate of unknown pathogen-host interactions. Thus, the truepositive rate of the predictions was expected to increase.In order to perform this study extensively, encoding methods and learning algorithms of several proteins were tested. Along with human as the host organism, two different pathogen organisms were used in the experiments. For each combination of protein-encoding and prediction method, both the original prediction algorithms were tested using only pathogen-host interactions and the same methodwas testedagain after integrating the known protein interactions within each organism. The effect of merging the networks of pathogen-host interactions of different species on the prediction performance of state-of-the-art methods was also observed. Successwas measured in terms of Matthews correlation coefficient, precision, recall, F1 score, and accuracy metrics. Empirical results showed that integrating the host and pathogen interactions yields better performance consistently in almost all experiments.

## 1. Introduction

Infectious diseases, such as HIV, Influenza, SARS, and COVID-19 are caused by viral and bacterial infections and affect the health of millions of people, and even lead to deaths each year. For example, infectious diseases resulted in 9.2 million deaths in 2013, accounting for about 17% of all deaths (Naghavi et al., 2015). In addition to affecting human health, it results in major economic losses. New coronavirus disease (COVID-19) has spread to many countries and is declared as a pandemic by the World Health Organization. According to OECD studies, the world economy is expected to contract by at least 2.4% in 2020Organisation for Economic Co-operation and Development (OECD) (2020). OECD Interim Economic Assessment Coronavirus: the world economy at risk [online]. Website https://www.oecd.org/berlin/publikationen/Interim-Economic-Assessment-2-March-2020.pdf [accessed 02 March 2020].. According to UNCTAD, by the end of 2020, foreigndirect investment flowsare expected to decrease by 30%–40%United Nations Conference on Tradeand Development (UNCTAD) (2020). Impact of the COVID-19 Pandemic on Global FDI and GVCs–Updated Analysis [online]. https://unctad.org/en/PublicationsLibrary/diaeiainf2020d3_en.pdf [4 March 2020].. ILO foresees that COVID-19 pandemic could increase global unemployment by almost 25 million by 2020International Labour Organization (ILO) (2020). ILO Monitor: COVID-19 and the world of work (2nd ed.) [online]. Website https://www.ilo.org/wcmsp5/groups/public/@dgreports/@dcomm/documents/briefingnote/wcms_740877.pdf [accessed 07 April 2020].. 

One key characteristic of infectious diseases is that the proteins of the pathogen organism interact withthe host organism’s proteins and influence their functionality. Understanding the mechanism that governs such interactions between the host and pathogenic organisms is of utmost importance in developing treatment strategies.

Existing studies on protein interactions can be considered in two categories. The first one explores the interactions of proteins within a species (Mei, 2013). These studies model the collection of interactions as protein-protein interaction (PPI) networks. Such networks has already been successfully used to understand the functions of proteins and the biological processes controlling vital functions of the cell and the results have been published in literature (Wu et al., 2006; Shen et al., 2007; De Bodt et al., 2009). The second one analyses the interactions of proteins across species. Such interactions are called pathogen-host interactions (PHI). Studying interspecies interactions has great potential to improve our understanding of the infection mechanism and thus leads to better treatment procedures. That said, most of the existing publications on protein interaction belong to the first category. As a result, although there are numerous resources for protein interactions within a species, the knowledge basedon interspecies interactions is limited. 

The methods used to determine protein interactions within or across species can be grouped into two categories: in vitro and in silico. In vitro methods can further be considered in two classes, namely small-scale and large-scale. The former one examines one protein pair at a time through genetic, biochemical, or biophysical experiments (Kshirsagar et al., 2013a). These methods typically take long time and require costly experimentation. In recent years, large-scale methods have been developed to detect thousands of protein interactions within a single experiment (Qi et al., 2010). Methods such as yeast two-hybrid systems, mass spectrometry and protein chip belong to this category. In vitro methods are expensive and time consuming. Thus, experimental testing of all possible combinations of protein pairs across organisms is not feasible as the number of such pairs can be massive. For example, exploring the interactions between a pathogenic organism that has 1000 proteins with about 100,000 proteins in human require 108 experiments. As a result, only a small fraction of possible interactions has been found using these methods. Experimentally verified interaction data are shared through databases such as Patric (Wattam et al., 2017), VirusMentha (Calderone et al., 2014), VirHostNet (Guirimand et al., 2015), PHISTO (Durmuş Tekir et al., 2013), and STRING (Szklarczyk et al., 2016).

The difficulty of applying in vitro methods to model interactions between proteins has promoted the development of computational methods. These methods use features such as protein structure, domain, gene neighbourhood, phylogenetic profiles, gene expressions and literature mining to predict interactions (You et al., 2015). Existing studies on computational methods are discussed in Section . These methods, however, have very low true positive rate, and thus miss significant fraction of true interactions.

The purpose of this study is to increase the true positive rate in predicting interactions between the proteins of a pathogen and a host organism. In this paper, it is presumed that protein interactions within an organism follow similar characteristics as those across organisms. Protein interactions within organisms are well-studied in the literature. There is a massive amount of available interaction data that are produced experimentally and computationally. String (Szklarczyk et al., 2016), KEGG (Kanehisa et al., 2017) and IntAct (Orchard et al., 2013) are a few examples of existing databases. Based on the assumption that is mentioned above, known intraspecies protein interaction networks of host and pathogen organisms were integrated to predict interspecies protein interactions. Yersinia pestis and Bacillus anthracis datasets were used as the pathogen organism models and human proteins as the host model. A strategy was developed to extend a suite of existing machine learning algorithms to integrate intraspecies interactions. These algorithms require a negative and a positive class of interactions. The negative class was generated by selecting pairs of proteins randomly; one from the host and the other from the pathogen organism that no known interaction exists. Three positive classes of interactions which are between (i) two pathogen proteins, (ii) two host proteins, and (iii) one pathogen and one host protein were selected. The known interactions were used in the String database as the positive samples in the first two classes. The positive sample for the third class was obtained from the PHISTO database. The host and pathogen proteins were encoded using three alternative sequence-based feature extraction methods. The assumption made was tested using six classification methods which appear widely in the literature, namely Bayesian network, naive Bayes, j48, K-star, kNN and random forest methods. In addition, these methods were tested on a new dataset where the interactome of two pathogen organisms was combined with the host organism to evaluate the impact of the assumption on the multitask learning problem. The performance of each method was evaluated in terms of accuracy, precision, recall, MCC and F1 scores. Experiments demonstrated that the proposed method increases the accuracy of true positive predictions dramatically. It was observed that integrating intraspecies protein interaction yields higher precision, recall, and thus F1 score in almost all combinations of datasets, classifiers, and feature selection methods.

The rest of the paper is organized as follows: Section presents the background needed to discuss our method. The datasets and our method are described in Section . Experimental results are presented in Section . Finally, the paper is concluded with a brief discussion in Section .

## 2. Background and preliminaries

In silico methods have been developed to model PPI since the interactions verified by in vitro methods cover a scant portion of all possible interactions. (Zhou et al., 2013) and (Nourani et al., 2015) presented comprehensive reviews ofin silico methods used in PHI estimation. In silico methods can be classified by machine learning, homology, structure, domain, and motif-based approaches as stated in these reviews. Data scarcity, data unavailability, and negative data sampling constitute the three major problems for all of these computational approaches (Mei, 2013).

 Supervised and semisupervised machine learning methods are used in many studies to solve the PHI problem (Baldi and Brunak, 2001; Bock and Gough, 2001). Supervised learning methods need a sufficient number of labeled samples for the prediction of each class. In order to solve the PHI problem with supervised learning methods, the positive (interacted) and negative (noninteracted) labeled data must be present in the dataset. In vitro methods provide experimentally verified data which are regarded as positive samples. However, it is not possible to access any experimentally verified non-interacted protein pairs. The absence of the validated negative samples is called the negative data sampling problem in supervised methods. Hence, the construction of the negative samples is a problem that must be overcome in the PHI prediction with supervised methods. Some studies present data mining methods which use only positive samples to build a prediction model (Mukhopadhyay et al., 2010; Mondal et al., 2012; Ray et al., 2012). Since the data mining methods use only positive samples, the model fails to predict negative interactions and so they have risk of high false positive rate.

In most of the studies that use both positive and negative samples, a noninteracted class is generated by selecting proteins randomly from pathogen and host (Bock and Gough, 2003; Martin et al., 2005; Nanni, 2005). When compared with all possible interactions between the host and pathogen proteins, the number of noninteracted protein pairs is scarce. Therefore, the probability of randomly selected pairs belonging to the positive class is very low. The ratio of the positive class to the negative class varies in studies. For instance (Mei, 2013) used equal number of classes, while (Kshirsagar et al., 2016) used 1:100 ratio. (Mei, 2013) separated subcellular colocalized pairs from noninteracted samples, and reported better performance. Dyer et al. (2011) investigated the effect of the positive to negative ratio on a classification in their study. They compared the accuracy results for 10 datasets containing different numbers of negative samples and reported that the percentage of the negative samples in the entire dataset does not have a considerable effect on the accuracy results.

Another problem encountered in PHI estimation is data scarcity. Multitask methods, that allow the use of interactions of more than one species, have been developed to overcome the data scarcity problem in pathogenic systems. Multitask methods use commonalities among different domains and learn problems simultaneously within a shared task formulation. (Nourani et al., 2015) and (Qi et al., 2010) proposed a semisupervised multitask method to predict PHI from a partially labeled dataset. Kshirsagar et al. (2013b) developed a task regularization-based framework that incorporates the similarities in biological pathways targeted by various diseases. Xu et al. (2010) used a collective matrix factorization based approach Kshirsagar et al., (2016) presented a multitask matrix completion to the multitask setting incorporating the structures of the tasks and providing a mechanism to share information between them.

## 3. Datasets and methods

In this section, first a short description of the datasets that are used in this study is provided. Then, the description of the extended network model (ENM) is presented. Location-based encoding (LBE) (Kösesoy et al., 2019), amino acid pairs (AAP) (Chen et al., 2007), and amino acid composition (AAC) (Bhasin and Raghava, 2004) are used for feature encoding. All the encoding methods are sequence-based and generate a fixed size feature vector independent of amino acid sequence length. Six prediction methods are used: random forests (Breiman, 2001), j48 (Bhargava et al., 2013), kNN (Dasarathy, 1991), naïve Bayes (Muralidharan and Sugumaran, 2012), Bayesian networks (Friedman et al., 1997), and K-star (Cleary et al., 1995). The details of encoding and prediction methods are given as appendices in the supplementary material section (Appendices A and B).

**Figure 1:**
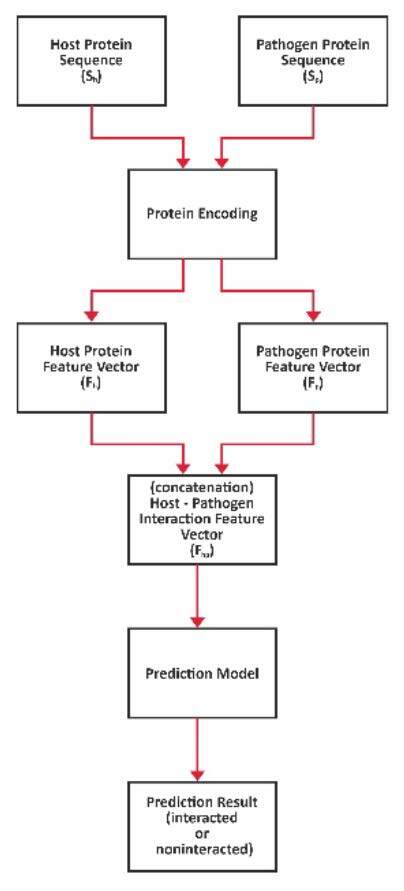
Steps of host-pathogen interaction prediction.

The final objective of this paper is to predict the interaction status (the response of the model is either “interacted” or “noninteracted”) of two proteins that belong to the host and pathogen organisms, respectively. To do this, first each protein’s amino acid sequence is encoded and the numeric feature vector is generated. Proteins are encodedby AAC, AAP and LBE methods. Then, these feature vectors are concatenated, and the final feature vector that is needed for the prediction model is acquired. The steps of the host-pathogen interaction predictionare displayed in Figure 1.

### 3.1. Datasets

In this work, the interaction data of Bacillus anthracis and Yersinia pestis pathogenswere used with human proteins to test the presented method and to compare it with available hitherto methods in the literature. Two sets of PHIs were obtained from PHISTO, which is a web accessible database extracting PHI data from nine databases and presenting interactions between data in a consistent format. While the PHI data are sufficient for the implementation of the methods in the literature, our method needs also intraspecies interaction network of proteins located in the related PHI network. The intraspecies PPIs were downloaded from the STRING database and the negative class of the species was constructed from UniProt database. The interaction data downloaded from the STRING database were filtered according to the combined score,which is calculated from features such as experimentally determined interaction, automated text mining database annotation, coexpression, etc.The combined score thresholds are given in Table 1.

**Table 1:** The combined score thresholds for datasets downloaded from STRING.

	B. anthracis	Yersinia pestis
Host-host int.	0.913	0.923
Pathogen-pathogen int.	0.704	0.974

The number of proteins and interactions of Yersinia pestis and Bacillus data used in this study were given in Table 2. Human proteins were used as host for both sets of data. The results become biased if homologous samples exist in the test and train sets at the same time. To avoid this issue, the similarity of sampleswere examined by using distance matrix;the distances were calculated for each sample and a lookup table was prepared for interaction data. With the lookup table (the datasets and lookup tables can be found in supplementary files), the test and training data were prevented to be similar. The lookup table is a symmetric square matrix showing the distance of each protein to the others. BLOSUM-62 scoring matrix was used for alignment and p-score value was calculated for distance. The p-distance is close to 1 for poorly related sequences and it is close to 0 for similar sequences. The threshold value was chosen as 0.7 for minimum sequence similarity between the samples. Consequently, none of the protein pairs in the dataset shared more than 30% sequence identity at any point of the validation procedure.

**Table 2:** Number of PH and HH interactions obtained from the PHISTO and STRING databases.

	B. anthracis	Yersinia Pestis
# of known PH interactions	3050	4097
# of used negative PH interactions	9500	12950
# of used PH interactions	1900	2590
# of used HH int.	1500	2000
# of used PP int.	234	176

To hinder bias on the extended datasets, the number of interspecies positive sampleswas reduced in the datasets. Our criterion to select the positive samples in the datasets is to have higher interaction possibility given by the STRING database. That is, for Bacillus pathogen 1500 distinct positive sampleswere used in the HH-dataset and likewise 234 positive samples were used in the PP-dataset in interaction network. For Yersinia pestis pathogen, 2000 distinct positive samples were used in the HH-dataset and 176 positive samples in the PP-dataset.

Noninteraction data were constructed by selecting negative protein pairs randomly from all possible —separating known ones— interactions. The number of random pairs chosen as the negative class was decided depending on the interaction rate. Choosing a ratio of 1:100 means that 1 in every 100 random pathogen-host protein pairs is expected to interact (Kshirsagar et al., 2013b). In an adjacency matrix, which shows the interactions between the proteins of two organisms, number of the known interactions (where set to 1) are sparse. Thus, in the dataset, the number of negative samples should be greater than the number of positive samples.We incorporate the prior on the interaction ratio by setting the size of our randomly sampled negatives equal to 5 times the number of positives.

The dataset, which was formed after all these pre-processing steps, was used in the experiments. 10-fold cross validation (CV) method was used to evaluate the classifiers tested in this paper. To do this, the dataset was divided into 10 equal sized subsets randomly. Nine of them were used for training and the remaining one for testing. This was applied by using each of the 10 subsets as the test class.

### 3.2. Extended network model

In this study, our objective is to increase the true positive ratio in the PHI prediction by considering the data scarcity, data unavailability and negative data sampling, which are the major problems encountered in the PHI estimation (Mei, 2013). To this end, besides the PHI, the interaction networks of both species were also included in the learning process.

**Figure 2:**
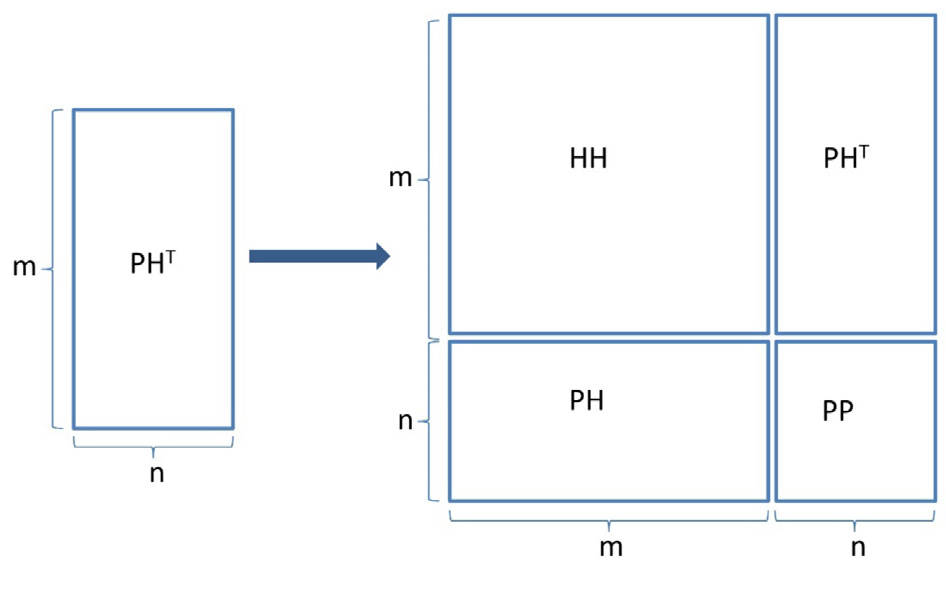
Single task data extension.

Let X=(*x_1_*,*x_2_*,...,*x_m_*) be the feature vector of m host proteins and Y=(*y_1_*,*y_2_*,...,*y_n_*) be the feature vector of n pathogen proteins. Let G be a bipartite graph connecting nodes of X and Y. And let Ω be (*x_i_*, *y_i_*), the set of all negative and positive classes of interactions. The links in the graph G can be represented by an *m x n* adjacency matrix (AM), M ∈ *R^mxn^*. The known interactions M were set to 1 and unknowns to 0 in the AM. The AM was extended in this method by merging the intraspecies interactions with PHI. The new AM, M ∈ *R^kxk^* and k = m + n , is a symmetric, square matrix with the dimensions of k x* k* as in Figure 2. In this case, the new set of all observed edges, Ω^*new*^, consisted of host-host (HH), pathogen-host (PH), and pathogen-pathogen interactions as follows:

1Ω1={(xi,,yj)},PH int.

2Ω2={(xi,,xj)},i≠j,HH int.

3Ω3={(yi,,yj)},i≠j,PP int.

4Ωnew=Ω1,∪Ω2,∪Ω3

Equations 2 and 3 show the intraspecies interactions, while Equation 1 shows pathogen-host protein interactions. The edge list of PH interactions, Ω_1_, contains also probable negatives. Other edge lists, (Ω_1_ *and* Ω_1_) , were generated based on the network of interactions downloaded from the STRING database and consist of only known interactions. While the datasets were merged, attention was paid to the total number of intraspecies interactions to be equal with the number of PHIs. The intraspecies interactions can be very large, especially when the human proteins are chosen as the host; therefore, such a case might cause an over-fit in the learning process. To hinder bias on the datasets, the positive samples tested in the datasets were reduced. Our criterion to select the positive samples in the datasets is to have higher interaction possibility given by the STRING database (see Section for details). The interaction result was filtered according to the combined score which is provided in the STRING. 

**Figure 3:**
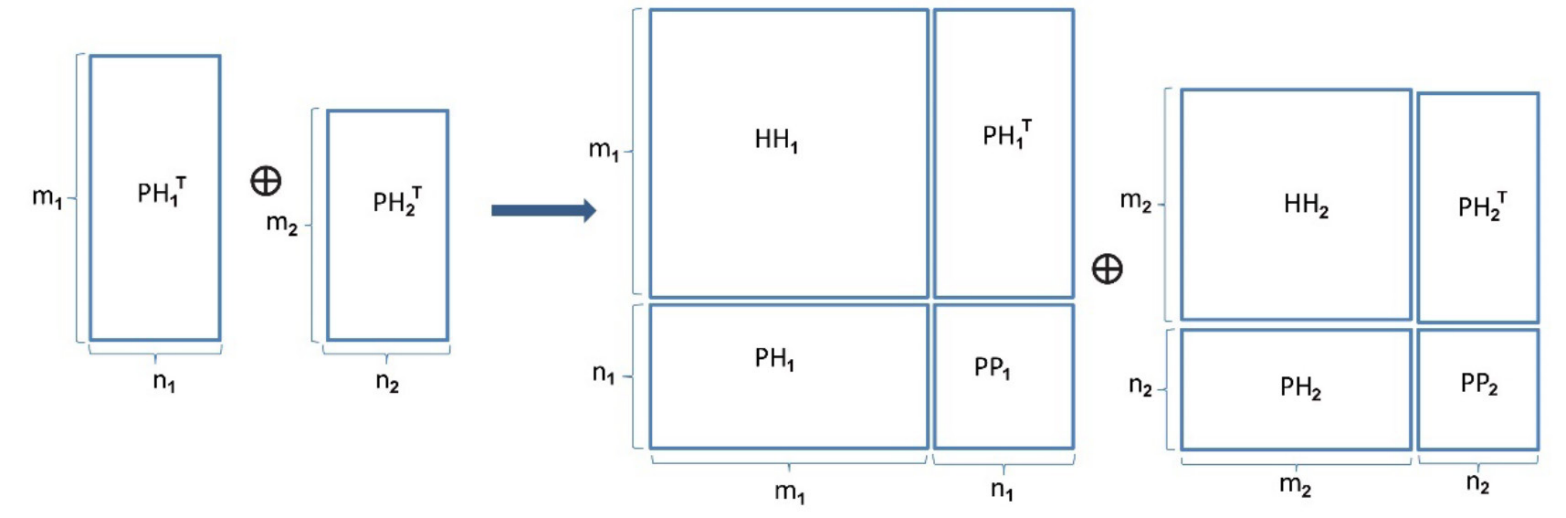
Multitask data extension.

In Figure 3 the integration of multiple pathogens is shown along with their interactions according to the ENM. In Section the impact of combining Yersinia pestis and Bacillus anthracis datasets is evaluated. 

## 4. Results

In this section, our method is evaluated experimentally. Two pathogen organisms (Yersinia pestis and Bacillus anthracis) and human, as the host organism, were used in our experiments (see Section for dataset details). The impact of our assumption, that integrates intraspecies interactions for predicting pathogen-host protein interactions, was measured on six well known methods, namely Bayesian networks, naive Bayes, random forest, J48, kNN, and K-star. The success/failure of our method was evaluated based on five measures, namely Matthews correlation coefficient (MCC), F1, precision, recall, and accuracy. In the following parts, these measures are explained thoroughly.

**Figure 4:**
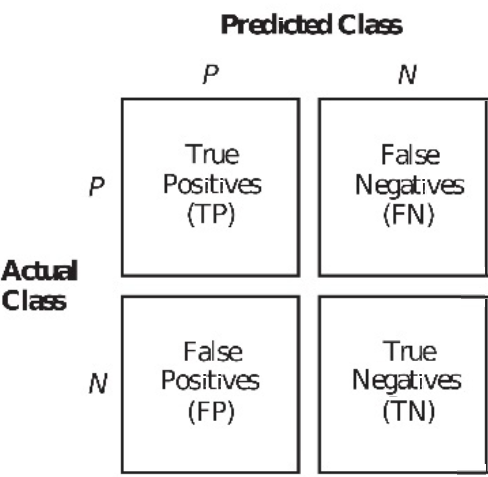
Confusion matrix.

The measures that are used in our experiments were derived from a 2×2 matrix called the confusion matrix (Davis and Goadrich, 2006). Confusion matrix shows the relationship between the predicted and actual classes. Figure 4 illustrates the concept of confusion matrix. Each entry in this matrix shows the number of samples falling into the corresponding (actual, predicted) class pair. Using this matrix, the measures were computed as follows:

5Precision=TPTP+FP

6Recall=TPTP+FN

7F1=2xPrecisionxRecallPrecision+Recall

8Accuracy=TP+TNTP+FP+TN+FN

9MCC=TPxTN-FPxFN(TP+FP)(TP+FN)(TN+FP)(TN+FN)

A result with high precision indicates that the predictions of the model on positive class are successful. High recall means that the model predicts most of the true interactions, yet it may predict false interactions in addition to them. The F1 score combines the two previous measures as their harmonic mean. High F1 score implies that both the precision and recall values are high. Thus, the F1 score gives a better understanding of the evaluation of the performance of a classification model than precision and recall alone.

10-fold cross validation (CV) was used to evaluate the classifiers tested in this paper. To do this, the dataset was divided into 10 equal sized subsets randomly. Nine of them were used for training and the remaining one for testing. This was applied by using each of the 10 subsets as the test class. The average value of the evaluation metrics observed was reported in all 10 experiments. Weka software (Hall et al., 2009) was used to test all the learning algorithms. The feature vector extraction step was implemented in MATLAB and PROSES web server (Kösesoy et al., 2018).

### 4.1. Evaluation of pathogen-host interactions

In our first experiment, the main hypothesis presented in this paper, that integrating the known protein interactions within host and pathogen organisms to improve the prediction success of unknown pathogen-host interactions, was tested. For each combination of protein-encoding and prediction method, both the original prediction algorithms were tested using only pathogen-host interactions and the same method after integrating the known protein interactions within each organism. For each method, the success was measured in terms of five criteria, namely precision, recall, F1 score, MCC, and accuracy. Tables 3 and 4 present the results using Bacillus and Yersinia as the pathogen models, respectively. Human was used as the host model.

**Table 3:** The evaluation results for Bacillus anthracis dataset.

		PHI					ENM				
Feat.	Meth.	Prec.	Rec.	F1	MCC	Acc.	Prec.	Rec.	F1	MCC	Acc.
AAC	BN	0.453	0.663	0.538	0.437	0.811	0.661	0.776	0.714	0.596	0.828
	NB	0.325	0.735	0.451	0.331	0.702	0.532	0.784	0.634	0.474	0.75
	kNN	0.396	0.639	0.489	0.373	0.777	0.593	0.818	0.688	0.556	0.794
	K-star	0.379	0.736	0.501	0.395	0.755	0.572	0.874	0.691	0.565	0.784
	j48	0.458	0.417	0.437	0.331	0.821	0.706	0.693	0.7	0.586	0.835
	RF	0.866	0.303	0.449	0.472	0.876	0.956	0.634	0.762	0.717	0.891
AAP	BN	0.417	0.707	0.525	0.422	0.787	0.598	0.675	0.634	0.485	0.785
	NB	0.495	0.421	0.455	0.359	0.832	0.727	0.439	0.547	0.451	0.799
	kNN	0.652	0.466	0.543	0.479	0.869	0.839	0.692	0.758	0.683	0.878
	K-star	0.643	0.512	0.57	0.502	0.874	0.727	0.636	0.678	0.602	0.873
	j48	0.518	0.503	0.51	0.415	0.839	0.713	0.728	0.72	0.612	0.844
	RF	0.827	0.386	0.527	0.515	0.884	0.916	0.688	0.786	0.732	0.896
LBE	BN	0.429	0.791	0.556	0.468	0.789	0.629	0.797	0.703	0.579	0.814
	NB	0.491	0.409	0.446	0.350	0.831	0.716	0.429	0.537	0.438	0.795
	kNN	0.638	0.513	0.569	0.498	0.87	0.807	0.737	0.77	0.689	0.878
	K-star	0.699	0.131	0.221	0.255	0.846	0.938	0.442	0.601	0.572	0.838
	j48	0.52	0.534	0.527	0.431	0.84	0.737	0.762	0.75	0.652	0.859
	RF	0.783	0.468	0.586	0.550	0.89	0.892	0.733	0.804	0.746	0.901

**Table 4:** The evaluation results for the Yersinia pestis dataset.

		PHI					ENM				
Feat.	Meth.	Prec.	Rec.	F1	MCC	Acc.	Prec.	Rec.	F1	MCC	Acc.
AAC	BN	0.407	0.639	0.497	0.384	0.785	0.608	0.743	0.668	0.535	0.802
	NB	0.303	0.683	0.42	0.284	0.685	0.473	0.741	0.578	0.393	0.708
	kNN	0.389	0.525	0.447	0.322	0.783	0.589	0.766	0.666	0.530	0.793
	K-star	0.416	0.683	0.517	0.411	0.788	0.597	0.835	0.696	0.575	0.804
	j48	0.464	0.416	0.439	0.335	0.823	0.684	0.674	0.679	0.563	0.829
	RF	0.954	0.27	0.421	0.469	0.876	0.973	0.575	0.723	0.695	0.881
AAP	BN	0.391	0.685	0.498	0.387	0.77	0.548	0.653	0.596	0.432	0.762
	NB	0.43	0.423	0.427	0.314	0.811	0.622	0.426	0.506	0.378	0.776
	kNN	0.596	0.366	0.454	0.389	0.853	0.811	0.635	0.713	0.632	0.862
	K-star	0.612	0.462	0.527	0.457	0.866	0.704	0.561	0.624	0.548	0.864
	j48	0.486	0.476	0.481	0.378	0.829	0.691	0.708	0.7	0.588	0.837
	RF	0.838	0.33	0.473	0.479	0.878	0.912	0.645	0.756	0.698	0.888
LBE	BN	0.395	0.778	0.524	0.429	0.764	0.576	0.792	0.667	0.531	0.787
	NB	0.414	0.401	0.408	0.292	0.806	0.602	0.387	0.471	0.344	0.766
	kNN	0.598	0.453	0.516	0.440	0.858	0.776	0.707	0.74	0.651	0.866
	K-star	0.677	0.154	0.251	0.272	0.847	0.903	0.445	0.596	0.559	0.838
	j48	0.5	0.505	0.502	0.402	0.833	0.707	0.728	0.718	0.612	0.846
	RF	0.79	0.406	0.536	0.503	0.883	0.883	0.7	0.78	0.720	0.894

Our results support our hypothesis. They demonstrate that integrating the host and pathogen interactions consistently yields better F1 scores in all 36 experiments of the protein-encoding, prediction method, and dataset combinations. Furthermore, the gap between the F1 score of extended network model (ENM) and that of PHI is dramatically high in almost all the experiments. Focusing on the two parameters which play an important role in the F1 score (i.e. precision and recall), it is observed that our method yields better precision and recall in nearly all experiments. More specifically, ENM has higher recall in 33 out of 36 experiments and higher precision in all experiments. 

Notice that unlike F1 score, precision, and recall values, PHI produces more accurate values than ENM in a few experiments. This is because protein interaction networks are sparse. For instance, consider the human Bacillus PPIs which have 907 pathogens and 1,568 host proteins. These two sets of proteins yield over 1.4 million protein pairs in total (i.e. 907 × 1568). However, there are only 3050 known interactions among all those protein pairs. That means only 0.2% of the protein pairs are known to interact between the host and pathogen. Therefore, the dataset is naturally biased towards the negative class. As a result, the accuracy measure is biased towards the negative class substantially. The discussion of the accuracy value was omitted in the rest of this paper for this reason. Next, each encoding technique will be investigated one by one.

Using AAC encoding, it is observed that ENM has better positive class prediction and a higher F1 score compared to PHI for all classifiers. RF produces the best F1 score for ENM on both the Bacillus and Yersinia datasets. BN produces the best F1 score for PHI 

on Bacillus dataset. K-star method yields the best F1 score for PHI on the Yersinia dataset. The results imply that ENM is stable and yields similar performance across different datasets as well as prediction methods. Overall, our results demonstrate that the relative success of ENM in terms of the F1 score remains similar among different measures on both datasets.

Next, the AAP encoding will be explained. Our results are similar to those in the AAC encoding except for PHI on Bacillus dataset. RF is slightly better than BN for PHI on Bacillus. However, RF produces the worst recall value on both the Yersinia and Bacillus datasets. ENM still has a higher F1 score than PHI on both datasets.

Using the LBE encoding, it is observed that ENM is superior to PHI in all experiment settings in terms of the F1 score. Our results are consistent with the two previous encodings. RF produces the best scores for ENM. One of the remarkable results in the tables is that the K-star method has very low values on both datasets. BN produces the best F1 score for PHI on both datasets. Furthermore, the gap between the F1 score of BN and the other prediction methods is dramatically high.

Notice that the two datasets, Bacillus and Yersinia, are different in terms of the number of protein interactions in the pathogen network (see Table 2). Despite such difference in dataset characteristics, ENM remains to yield high F1 scores. This suggests that ENM is also stable across different dataset sizes. Overall, it is concluded that ENM is superior to PHI across a wide spectrum of prediction methods, feature encoding strategies, and dataset characteristics. It is also robust as it consistently produces accurate results.

### 4.2. Evaluation of the integration of multiple pathogens

In the second experiment, the impact of combining multiple pathogens, along with their interactions with a given host organism, on the success/failure of the predictive power of PHI was evaluated. Yersinia and Bacillus were used as the pathogen models and human was used as the host organism model. The same three protein-encoding techniques were used, and the six prediction methods were employed in these experiments as in the previous section and the results were presented by the same five success criteria. Table 5 presents the results. 

**Table 5:** The evaluation results for the merged dataset.

		Merged dataset				
Feature	Method	Prec.	Rec.	F1	MCC	Acc.
AAC	BN	0.412	0.648	0.504	0.393	0.788
	NB	0.304	0.693	0.423	0.289	0.685
	kNN	0.4	0.598	0.479	0.360	0.783
	K-star	0.411	0.733	0.526	0.426	0.78
	j48	0.495	0.434	0.462	0.365	0.832
	RF	0.926	0.306	0.46	0.489	0.88
AAP	BN	0.398	0.687	0.504	0.395	0.775
	NB	0.447	0.412	0.428	0.320	0.817
	kNN	0.633	0.412	0.499	0.437	0.862
	K-star	0.627	0.51	0.562	0.491	0.872
	j48	0.504	0.496	0.5	0.401	0.835
	RF	0.831	0.36	0.502	0.499	0.881
LBE	BN	0.407	0.787	0.536	0.445	0.773
	NB	0.444	0.394	0.417	0.310	0.817
	kNN	0.617	0.474	0.536	0.463	0.863
	K-star	0.693	0.148	0.244	0.271	0.847
	j48	0.522	0.53	0.526	0.430	0.841
	RF	0.793	0.435	0.562	0.528	0.887

Among all combinations of protein-encoding and prediction methods, the highest F1 score was obtained using LBE, and BN together. Also, BN method yields the highest F1 score for AAP encoding. When the results in Table 5 are compared with those in Tables 3 and 4, it is noticed that combining multiple pathogens does not improve the success rate of predictions. Typically, the F1 score of the combined dataset is between those of the individual datasets. For instance, while using AAC as the encoding method and BN as the prediction method, the F1 score of PHI, for a system of Yersinia and Bacillus together, becomes 0.504. While using only Bacillus and only Yersinia pestis, it becomes 0.538 and 0.497, respectively. In some experiments, it is even observed that combining the two pathogens decreases the F1 measure over both individual pathogens when they are considered separately (see AAP/NB combination). In this work, several possible underlying reasons are assumedto clarify these results. One of them is the variation between the amino acid sequences (and thus the feature vectors) across different pathogens. Another possible reason is the significant variation in the amount of interaction data available for the two pathogens. This may create biased learning towards the pathogen with more known interactions. Third reason is having very limited information on host-pathogen interactions currently. As such interaction data become available for more pathogens, it is anticipated that integrating multiple pathogens, particularly phylogenetically close pathogens, has a potential to further improve the prediction accuracy.

Also, further studies in balancing such variation (such as weighting the features obtained from different pathogens) have the potential to improve the prediction.

**Figure 5:**
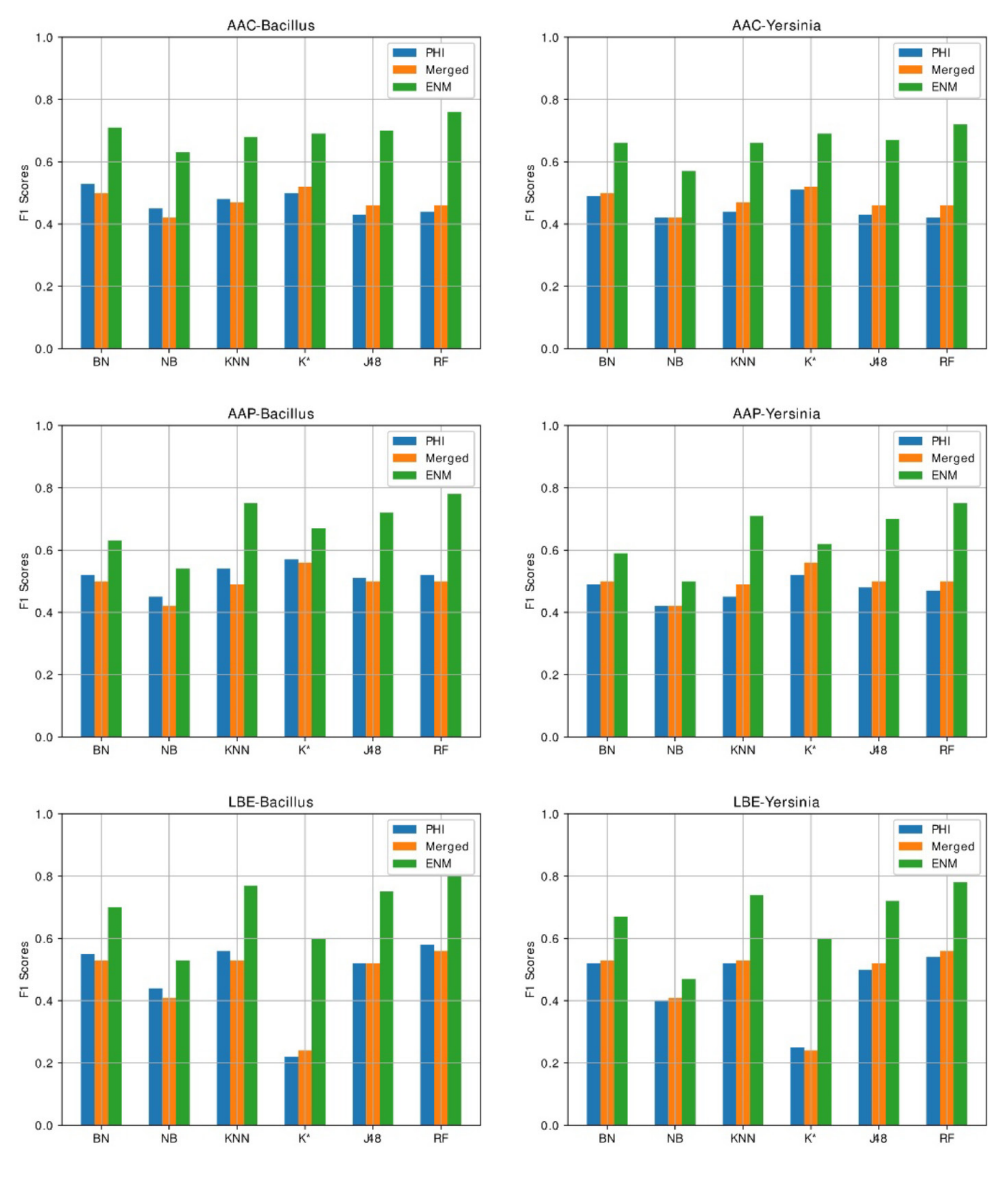
F1 scores of all experiments.

Figure 5 displays the graphical representation of the F1 results obtained from encoding and prediction method combinations. The ENM outperforms the PHI and merged dataset results in all experiments.

## 5. Conclusion

Data scarcity, data unavailability, and negative data sampling are three major problems in PHI estimation. The amino acid sequences are the most available data for both the host and pathogen organisms. Thus, a PHI prediction model that depends on only amino acid sequences has a great importance. Even though the amino acid sequence is the most available data among other protein features, interaction data are still scarce to train a robust prediction model. In this study, ENM was proposed especially to get over the data scarcity and data unavailability problems. Machine learning methods were used with diverse protein sequence encoding methods to predict the interactions between the host and pathogen proteins. We have achieved to increase the accuracy of prediction including intra-species interaction networks of host and pathogen in the learning process. It is observed that merging the PHI networks of different species tends to increase the performance of our method. That is, the first experiment shows that integrating the host and pathogen interactions consistently yields better F1 scores in protein-encoding, prediction method, and dataset combinations. In future work, our model ENM, can be extended to perform classification on multiclass labels. Additionally, we plan to develop a web server which is publicly available to implement ENM for other infectious diseases.

## Supplementary material

Supplementary materials associated with this article can be found at the following website: https://github.com/irfan7787/phiPrediction
